# Muscle-strengthening exercise and positive mental health in children and adolescents: An urban survey study

**DOI:** 10.3389/fpsyg.2022.933877

**Published:** 2022-08-15

**Authors:** Xiaohui Zhang, Chujuan Jiang, Xiaocong Zhang, Xinli Chi

**Affiliations:** ^1^School of Physical Education, Suzhou University, Suzhou, China; ^2^School of Art, Music and Dance, Shenzhen University, Shenzhen, China; ^3^Security Department, Suzhou University, Suzhou, China; ^4^School of Psychology, Shenzhen University, Shenzhen, China; ^5^Centre for Mental Health, Shenzhen University, Shenzhen, China

**Keywords:** muscle-strengthening exercise, subjective wellbeing, resilience, adolescents, physical activity, mental health

## Abstract

**Background:**

Much evidence has indicated that physical activity is associated with mental health benefits, such as fewer depression symptoms. Psychological resilience captures a broader mental health phenomenon that may be influenced by other factors as well. Yet, there are few studies examining the association between muscle-strengthening exercises (MSEs) with mental health outcomes, especially positive outcomes (e.g., subjective wellbeing). The study aimed to test the association between MSE with subjective wellbeing and resilience among a large sample of Chinese adolescents.

**Materials and methods:**

A cross-sectional study was conducted among primary and middle school students in Shenzhen, China. MSE frequency, subjective wellbeing, and resilience were measured. Data from a total of 67,281 children and adolescents aged 10–17 years (51.9% men and 48.1% women) were included in the analysis. Mixed effect models were performed to assess how weekly MSE frequency (0–7 days) related to the levels of subjective wellbeing and resilience, adjusting for potential confounding variables (e.g., sex and grade). Sensitivity analyses were also conducted.

**Results:**

Compared to participants with no MSE, the levels of subjective wellbeing were higher in those with higher MSE frequencies [range of β: 0.29 (1 day per week) to 1.98 (7 days per week)]. The frequency of MSE was also positively correlated to better resilience [range of β: 0.50 (1 day per week) to 4.40 (7 days per week)]. All associations remained significant in sensitivity analyses.

**Conclusion:**

More frequent MSE was associated with superior subjective wellbeing and resilience of Chinese children and adolescents. Increasing MSE may be beneficial for promoting positive mental health outcomes among children and adolescents.

## Introduction

Physical activity has become one of the most significant health-related aspects in the lives of children and adolescents (Belcourt et al., 2016). They are identified as closely related to individual mental health and psychological disease of children and adolescents (Biddle and Asare, 2011). Physical activities have immediate and long-term health benefits at the same time. Recently, researchers have focused on the different modalities of physical activity and their associations with health benefits (Moljord et al., 2014; Belcourt et al., 2016; Rhodes et al., 2017). Among the various different modalities of physical activity, muscle-strengthening exercise (MSE) (Buecker et al., 2021) is an important form of physical activity that has attracted sufficient research interest and much evidence has confirmed the health benefits of meeting the MSE guidelines (the guidelines recommend that young people should do three or more times of MSE per week). The relations between mental health and MSE have attracted the attention of many researchers and the public (Biddle and Asare, 2011). For example, Bennie et al. (2019) drew data from the U.S. 2015 Behavioral Risk Factor Surveillance System. His study stated the relation and associations of MSE with negative symptoms severity among adults in the United States of America. The previous studies demonstrated that depression has been a severe problem in modern society and has significant impacts on children’ and adolescents’ development or adults’ lives (Cohen et al., 2014; LaVigne et al., 2016; Warburton and Bredin, 2017). Studies also stated that both aerobic and MSE are associated with a lower likelihood of depressive symptoms severity (Gelaye et al., 2016; Bennie et al., 2019). Research also stated that physical activity is associated with indicators of sleep health (Gariépy et al., 2016; Werner-Seidler et al., 2017). The previous studies identified the significant functions of physical activity and MSE (Saunders et al., 2016; Yalcinkaya et al., 2017; Zasadzka et al., 2021). Mental health and subjective wellbeing overlap in concept, but can be regarded as different structures. Subjective wellbeing is usually defined according to the overall aspects of young people’s life wellbeing (Carson et al., 2016; Wang et al., 2020). Some studies indicate that subjective wellbeing is negatively correlated with emotional disorders (Liu et al., 2014) and externalizing behaviors (Andersen et al., 2017). The latest data from the Millennium Cohort study show the correlation between mental health problems and subjective wellbeing of school-aged students (Hwang et al., 2021). The concept of resilience originates from the observation of its results (Levis et al., 2019): in terms of psychological resilience, it refers to people’s health (or rapid recovery) in adversity (Levis et al., 2019). Psychological Resilience reflects a broader phenomenon of mental health, which may also be affected by other factors. It refers to the ability to maintain mental health or recover quickly after stress (Sun et al., 2021). Resilience is our “psychological immunity,” which comes from the interaction in the complex and multifaceted biological psychosocial system (Levis et al., 2019). The implication of this complex dynamic system is that elasticity can change from time to time and from environment to environment.

The noted evidence has suggested that participating in more MSE would be beneficial to mental health (Gelaye et al., 2016). Moreover, this study showed that while achieving both aerobic activity and MSE guidelines is promising in improving positive mental health (Plummer et al., 2016; Sun et al., 2021), such as health-related quality of life. But a number of limited studies have been replicated to examine the positive mental health benefits of MSE in adolescents (Biddle and Asare, 2011; Bennie et al., 2020b). It is expectedly possible that young people can gain mental health benefits from MSE. To our knowledge, only one study is examining the association between MSE and mental health disorders in young people. However, concerning the relationship between MSE and positive mental health outcomes, the relevant studies remain scanty. To fill the gaps in this literature, it is needed to explore the association between MSE and positive mental health outcomes in children and adolescents.

## Materials and methods

### Study design and participants

The data used in the current study came from a sampling survey performed in March 2021, and the large-scale survey was carried out in Shenzhen, one of the developed Chinese cities. By working with the Educational Science Research Institute of Shenzhen, a sampling design involving multiple stages was adopted in the present study, and local students from different middle and primary schools in Shenzhen were included in the study. All the schools included in the study are public schools, and they are supervised by the Shenzhen Education Bureau under the guidance of the Ministry of Education of the People’s Republic of China. The inclusion criteria include (1) grade 5 or 6 students of primary schools, or grade 1 or 2 students of junior high school, or grade 1 or 2 students of high school (the grade 3 students of junior high schools or high school, because they are busy with preparing for the college or high school entrance examination, and they can’t make time for the survey); (2) students with good reading and understanding ability; (3) the students who are willing to take the survey after being well informed of study details. The exclusion criteria included the students, their guardians, or the teachers who didn’t want them to take the survey or didn’t think it is suitable for them to take the survey. The questionnaire was posted on the Wenjuanxing platform,^1^ a platform for online survey in China. In this way, the students could conveniently fill out the form and submit it online. The purpose and background of the survey, and the informed consent were posted on the page before the questionnaire was started. All student participants and their teachers and guardians were informed of the details of the survey. The students who were willing to take the survey completed the form online (about 20 min to complete the form). Each class was a survey unit and the students would fill out the form during school time by following the instructions of their teachers under their supervision.

In total, 78,428 questionnaires were retrieved from 135 schools. After screening the questionnaires (for example, the questionnaires from the students who didn’t submit on time, or the questionnaires with unidentifiable answers, or with too many repetitive answers). A total of 67,821 valid questionnaires were retrieved from the students aged between 10 and 17 years used in the study. The collection of data had been approved by the Institutional Research Ethics Board of Shenzhen University (grant number: 2020005).

### Study measures

#### Muscle strengthening exercise

When collecting MSE information (Bull et al., 2020) about the students, the following questions were used: “In the last 7 days, how much time did you spend on the exercise to tone or strengthen the muscle, such as lifting weight, sit-ups, or push-ups?” The possible answers included: 0 = none, 1 = 1 day, 2 = 2 days, 3 = 3 days, 4 = 4 days, 5 = 5 days, 6 = 6 days, and 7 = 7 days. The item was adopted for surveillance of health behavior in other countries (Wiese et al., 2018). Moreover, the item was found to show acceptable reliability for children and adolescents with a Kappa coefficient greater than 0.55 (Kavetsos, 2011). Identical with the guidelines of the World Health Organization, children and adolescents who engaged in MSE for 3 days in the last 7 days had been considered as meeting the recommendations (Bottolfs et al., 2020).

#### Subjective wellbeing and resilience

Subject wellbeing is an indicator of hedonic wellbeing, and it was measured by the Five-item Wellbeing Index developed by the World Health Organization (Chinese version). Participants who had positive feelings in the past 2 weeks ranged from at no time to at all of the time on a 6-point scale. The higher the summed scores, the greater the levels of the subjective wellbeing. Psychometric properties of WHO-5 (Chinese version) were confirmed among adolescents in China, with a Cronbach’s α coefficient of 0.94 for WHO-5.

Resilience, an indicator of eudaimonic wellbeing, was measured by the Connor–Davidson Resilience Scale (Chinese version), also known as CD-RISC-10, which includes 10 items. The CD-RISC-10 includes response options and the answers are scored from 0 to 4. Here, “0” stands for never, while “4” stands for almost always. The higher the total score, the greater the resilience level. The CD-RISC-10 was found to be suitable for the survey on Chinese adolescents. The Cronbach’s α coefficient was 0.93 for CD-RISC-10 in Chinese adolescents.

#### Covariates

In addition to this, we also consider some sociodemographic factors as covariates in the further analysis, such as sex, age, grade, parental education level, perceived family affluence, and living with parents or not.

#### Statistical analysis

In the current study, all the statistical analyses were completed using STATA (BE/17.0 version). Descriptive statistics were used to report information on sample characteristics, which percentage was used for categorical variables while the mean was used for continuous variables. Two level-mixed effect models were performed to estimate the association between MSE and mental health indicators. Since the outcomes were tested as a normal distribution, adjusted beta coefficient (β) with corresponding 95% confidence intervals (95% *CIs*) were described. The lowest level of MSE was set as a reference for comparing the estimated results for outcomes to those with higher levels of MSE. In addition, to reduce the risk of estimation, we conducted a sensitivity analysis by stratifying MSE, of which were (1) 0–7 days; (2) 0–2 days, 3–5 days, and 6–7 days based on the previous studies (Bennie et al., 2020b); (3) meeting or not meeting the MSE guidelines according to the physical activity guidelines (Bull et al., 2020).

## Results

The sample consisted of 51.9% men and 48.1% women, with a mean age of 13.04 years. The proportion of participants in each grade was 41.5, 40.3, and 18.10%, respectively. Approximately 76.2% of the participants perceived their families in middle economic status, and 72.5% of participants were in normal body mass index status. More than half of the participants had MSEs for 0–2 days, namely not meeting the MSE guideline. More detailed sample characteristics are provided in Table 1.

**TABLE 1:** Sample characteristics in this study.

Categorical variables	Proportion (%)
Sex	
Male	51.9
Female	48.1
Siblings	
Only child	25.8
Non-only child	74.2
Father’s educational level	
Junior middle school or below	21.7
High school or equivalent	27.0
Bachelor or equivalent	38.7
Master or above	4.2
Unclear	8.4
Mother’s educational level	
Junior middle school or below	26.2
High school or equivalent	27.8
Bachelor or equivalent	35.6
Master or above	2.4
Unclear	8.0
Living	
Living with parents	93.4
Not living with parents	6.6
Subjective economic status	
Low	17.9
Middle	76.2
High	5.9
Grade	
Primary school	41.5
Junior middle school	40.3
High school	18.1
Body mass index	
Normal	72.5
Overweight	12.3
Obese	15.2
Muscle-strengthening exercise days (continuous)	
0 days	24.9
1 day	18.1
2 days	18.4
3 days	15.9
4 days	6.7
5 days	7.9
6 days	1.6
7 days	6.4
Muscle-strengthening exercise days (3 cut-offs)	
0–2 days	61.4
3–5 days	30.6
6–7 days	8.0
Muscle-strengthening exercise guideline	
Not meet	61.4
Meet	38.6
Age	13.04
Subjective wellbeing scores	20.15
Resilience scores	34.61

The association between MSE and subjective wellbeing scores is clearly presented in Table 2. Identifying the MSE day as a continuous variable, conducting MSE 1–7 days was positively associated with greater subjective wellbeing scores compared to exercise 0 day. Conducting MSE 3–5 days [β = 0.77, (0.67, 0.86)] and 6–7 days [β = 1.58, (1.43, 1.74)], was positively associated with greater subjective wellbeing scores compared to exercise 0–2 days when identifying exercise day as a 3 cut-offs variable. A positive association between meeting the MSE guidelines and greater subjective wellbeing scores [β = 0.93, (0.84, 1.02)], was observed compared to not meeting MSE guidelines.

**TABLE 2:** Results for the associations between muscle strengthening exercise and subjective wellbeing scores.

Subjective wellbeing scores	β	95%CI		*P*-value
Muscle strengthening exercise days (continuous)				
0 days	Ref			
1 day	0.29	0.16	0.42	0.000
2 days	0.63	0.50	0.76	0.000
3 days	0.86	0.73	1.00	0.000
4 days	1.20	1.01	1.38	0.000
5 days	1.37	1.20	1.55	0.000
6 days	1.50	1.16	1.84	0.000
7 days	1.98	1.80	2.17	0.000
Muscle strengthening exercise days (3 cut-offs)				
0–2 days	Ref			
3–5 days	0.77	0.67	0.86	0.000
6–7 days	1.58	1.43	1.74	0.000
Muscle strengthening exercise guidelines				
Not meet	Ref			
Meet	0.93	0.84	1.02	0.000

β, beta coefficient; CI, confidence interval; Ref, reference group.

It is shown clearly in Table 3 that conducting MSE 1–7 days, when exercise day was identified as a continuous variable, was positively associated with greater resilience scores compared to exercise 0 day. When identifying MSE day as a 3 cut-offs variable, conducting MSE 3–5 days [β = 1.64, (1.51, 1.78)] and 6–7 days [β = 3.60, (3.38, 3.83)], was positively associated with the greater resilience scores compared to conducting exercise 0–2 days. A positive association between meeting MSE guidelines [β = 2.04, (1.91, 2.17)] and greater resilience scores was observed when compared to not meeting MSE guidelines.

**TABLE 3:** Results for the associations between muscle strengthening exercise and resilience scores.

Resilience scores	β	95%CI		*P*-value
Muscle strengthening exercise days (continuous)				
0 days	Ref			
1 day	0.50	0.32	0.69	0.000
2 days	1.24	1.05	1.42	0.000
3 days	1.78	1.58	1.97	0.000
4 days	2.54	2.28	2.80	0.000
5 days	2.82	2.57	3.07	0.000
6 days	3.32	2.83	3.80	0.000
7 days	4.40	4.13	4.66	0.000
Muscle strengthening exercise days (3 cut-offs)				
0–2 days	Ref			
3–5 days	1.64	1.51	1.78	0.000
6–7 days	3.60	3.38	3.83	0.000
Muscle strengthening exercise guidelines				
Not meet	Ref			
Meet	2.04	1.91	2.17	0.000

β, beta coefficient; CI, confidence interval; Ref, reference group.

## Discussion

The present study investigated the associations of MSE with subjective wellbeing and resilience in children and adolescents from an urban city in China. We mainly found that more participation in MSE was favorably associated with subjective wellbeing and resilience scores in children and adolescents in this study. To our knowledge, our study is the very one first to explore whether MSE or not would be associated with better subjective wellbeing and resilience in children and adolescents. This study can advance the knowledge in mental health promotion. The below presents detailed discussions on the association between MSE and mental health indicators.

The previous study conducted that resistance training can effectively improve all areas of positive mental health (Bennie et al., 2019). Similarly, data showed that the mental health status of the strength training group was significantly improved after 12 weeks compared with the control group (Yu et al., 2021). Contrary to the results of current studies, some studies have pointed out that resistance training is effective in improving positive mental health (Kjaer, 1992; Smith et al., 2014; Yeatts et al., 2017). In addition, the previous studies have confirmed the positive association between physical activity and subjective wellbeing (Lubans and Cliff, 2011; Ortega et al., 2012; Rodriguez-Ayllon et al., 2018). In addition to this, there has been evidence suggesting sufficient physical activity role in promotion resilience in children and adolescents (Bottolfs et al., 2020). Such a research finding is replicated in other studies (Skrove et al., 2013; Moljord et al., 2014). Although evidence concerning the association between sufficient physical activity and subjective wellbeing and resilience is, at present relatively, the art of state in the literature can support the desirable effects of physical activity in improving mental health wellbeing indicators in children and adolescents. The above evidence can collectively support the current study. The sensitive analysis indicated that MSE was positively with both subjective wellbeing and resilience scores irrespective of cut-offs for the independent in children and adolescents. This result suggests that any increases in MSE would be a contributor of better mental health indicators. Because MSE is a specific type of physical activity, it is expected that MSE can play a similar role of physical activity, in terms of positive mental health promotion. In this regard, it would seem that promoting MSE for children and adolescents is a feasible approach for mental health interventions. Our study results maintain this expectation and provide cross-sectional evidence. Based on our research finding, it would be possible to increase positive mental health through MSE promotion in children and adolescents.

However, to better explain the association between MSE and subjective wellbeing and resilience, some possible underlying mechanisms can be proposed but need further supportive evidence. First of all, participation in physical activity can enhance adolescents’ wellbeing (Biddle and Asare, 2011). As MSE is a specific kind of physical activity, it is reasonable that participating in more MSE can enhance the mental wellbeing outcomes, such as subjective wellbeing and resilience. Another explanation is that MSE has been found to be negatively associated with mental health disorder indicators. For example, some observational studies have revealed an association between MSE and depression (Bennie et al., 2019, 2020a,2020b; Yu et al., 2021). Since mental wellbeing outcomes are conversely associated with the mental disorder indicators, it is sensible that MSE reduces the odds of mental disorder indicators, which in turn increase scores of mental wellbeing outcomes. However, this explanation needs further studies to examine, especially mediation studies. From the perspective of fitness promotion, participating in MSE at sufficient intensity can improve health-related fitness (i.e., muscular fitness), which may be necessary to induce neurobiological adaptations. According to the cross-stressor adaptation (CSA) hypothesis (Kjaer, 1992), participants who regularly engage in MSE have a higher response similar to that following exposure to a psychosocial stressor (i.e., increase in heart rate, respiration, and blood cortisol). The CSA hypothesis posits that the beneficial adaptation of the hypothalamic–pituitary–adrenocortical axis and the sympathoadrenal medullary system during physical exercise can generalize to psychosocial stressors and may improve positive mental health (Mücke and Ludyga, 2018).

Moreover, multiple studies using various muscle strength indices have linked MSE with mental health symptoms. It has been suggested that MSE may have unique mental health benefits for adolescents (Biddle and Asare, 2011; Smith et al., 2014; Yeatts et al., 2017). A recent study found that absolute upper- and lower-body muscular strength was positively associated with self-esteem in a small sample of overweight/obese children (Rodriguez-Ayllon et al., 2018). In another study involving adolescents, MSE was found to be positively associated with physical self-perceptions (i.e., perceived appearance and sports competence) (Lubans and Cliff, 2011), which are known to generalize to global self-esteem. The relationship between handgrip strength and perceived stress was dose-dependent for both sexes. Low handgrip strength was associated with poor mental health among boys (Hwang et al., 2021). These positive relationships support that MSE can improve the mental health through the better physical fitness levels. Powerful evidence for the benefits of MSE for mental health can be observed in a 24-year longitudinal study involving more than one million male adolescents (16–19 years) (Ortega et al., 2012). Another longitudinal study involving middle school students revealed that higher levels of physical fitness, such as MSE, were associated with improved mental health (i.e., lower levels of depression, peer rejection, loneliness, internalizing symptoms as well as higher levels of adaptive functioning, externalizing symptoms, self-worth, and perceived competence) (LaVigne et al., 2016).

## Study limitations and strengths

A key limitation is that the cross-sectional design limits inferences of causality for the association between MSE and mental outcomes. Longitudinal and experimental evidence related to this topic is scarce and future studies should adopt the longitudinal and experimental design to examine the findings from cross-sectional studies. Due to the age range of 10–17 years involved in this study, there may be some limitations. A further limitation concerns the measurement of MSE. Following the recommendation raised by the WHO, which only focuses on the frequency of MSE, we did not capture other aspects of MSE, such as duration and type. However, these factors might affect the association between the frequency of MSE and health outcomes. Future studies are encouraged to investigate more information about MSE and its potential health benefits. As for the strengths of this study, the large sample size is notable. More importantly, considering that most studies that aimed to examine the relationship between physical activity and mental health outcomes used negative outcomes or psychological symptoms (e.g., depression and anxiety), we set positive mental health indicators as the outcomes, which add new evidence to the existing knowledge base from another perspective. Meanwhile, in addition to demographic factors, depression and anxiety scores were adjusted in the models to better clarify the relationship between MSE and positive mental health outcomes. Besides, it should be noted that we used reliable and valid questionnaires to measure the mental outcomes.

## Conclusion

Among a large sample of Chinese children and adolescents, the weekly frequency (days) of MSE was associated with a higher level of subjective wellbeing and resilience. Although longitudinal and experimental studies are needed to better verify the association, our preliminary evidence suggests that at least 1 day of MSE might be related to improved positive mental health outcomes. Hence, MSE deserves more attention in physical activity programs targeted at children and adolescents. These findings highlight an urgent unmet need in future MSE public health intervention for children and adolescents. Moreover, the benefits of MSE on mental health may also be emphasized in health education.

## Data availability statement

The raw data supporting the conclusions of this article will be made available by the authors, without undue reservation.

## Author contributions

XHZ developed the screen strategy, drafted the manuscript, and summarized the findings. CJ contributed to methodology, summarized the findings, and drafted the manuscript. XCZ contributed to formal analysis, review, and editing and assessed the manuscript. XC contributed to resources, data curation, and analysis. XHZ and CJ reviewed and edited the final manuscript. All authors have read and agreed to the published version of the manuscript.

## Footnotes

1 https://www.wjx.cn/, accessed on March 8, 2022.

## References

[B1] Andersen J. R. Natvig G. K. Aadland E. Moe V. F. Kolotkin R. L. Anderssen S. A. (2017). Associations between health-related quality of life, cardiorespiratory fitness, muscle strength, physical activity and waist circumference in 10-year-old children: the ASK study. *Qual. Life Res.* 26 3421–3428. 10.1007/s11136-017-1634-1 28656535

[B2] Belcourt V. Gray C. Borghese M. Carson V. Chaput J.-P. Janssen I. (2016). Systematic review of the relationships between objectively measured physical activity and health indicators in school-aged children and youth applied physiology. *Nutr. Metab.* 41 S197–S239. 10.1139/apnm-2015-0663 27306431

[B3] Bennie J. A. Teychenne M. Tittlbach S. (2020b). Muscle-strengthening exercise and depressive symptom severity among a nationally representative sample of 23,635 german adults. *J. Affect. Disord.* 266 282–287. 10.1016/j.jad.2020.01.172 32056889

[B4] Bennie J. A. De Cocker K. Biddle S. J. H. Teychenne M. J. (2020a). Joint and dose-dependent associations between aerobic and muscle-strengthening activity with depression: a cross-sectional study of 1.48 million adults between 2011 and 2017. *Depression Anxiety* 37 166–178. 10.1002/da.22986 31876971

[B5] Bennie J. A. Teychenne M. J. De Cocker K. Biddle S. J. H. (2019). Associations between aerobic and muscle-strengthening exercise with depressive symptom severity among 17,839 U.S. Adults. *Prev. Med.* 121 121–127. 10.1016/j.ypmed.2019.02.022 30786252

[B6] Biddle S. J. Asare M. (2011). Physical activity and mental health in children and adolescents: a review of reviews. *Br. J. Sports Med.* 45 886–895. 10.1136/bjsports-2011-090185 21807669

[B7] Bottolfs M. Støa E. M. Reinboth M. S. Svendsen M. V. Schmidt S. K. Oellingrath I. M. (2020). Resilience and lifestyle-related factors as predictors for health-related quality of life among early adolescents: a cross-sectional study. *J. Int. Med. Res.* 48:0300060520903656. 10.1177/0300060520903656 32070172 PMC7111039

[B8] Buecker S. Simacek T. Ingwersen B. Terwiel S. Simonsmeier B. A. (2021). Physical activity and subjective well-being in healthy individuals: a meta-analytic review. *Health Psychol. Rev.* 15 574–592. 10.1080/17437199.2020.1760728 32452716

[B9] Bull F. C. Al-Ansari S. S. Biddle S. Borodulin K. Buman M. P. Cardon G. (2020). World Health Organization 2020 guidelines on physical activity and sedentary behaviour. *Br. J. Sports Med.* 54:1451. 10.1136/bjsports-2020-102955 33239350 PMC7719906

[B10] Carson V. Tremblay M. S. Chaput J. P. Chastin S. F. (2016). Associations between sleep duration, sedentary time, physical activity, and health indicators among Canadian children and youth using compositional analyses. *Appl. Physiol. Nutr. Metab.* 41 (6 Suppl. 3) S294–S302. 10.1139/apnm-2016-0026 27306435

[B11] Cohen D. D. Gómez-Arbeláez D. Camacho P. A. Pinzon S. Hormiga C. Trejos-Suarez J. (2014). Low muscle strength is associated with metabolic risk factors in Colombian children: the ACFIES study. *PLoS One* 9:e93150. 10.1371/journal.pone.0093150 24714401 PMC3979680

[B12] Gariépy G. Honkaniemi H. Quesnel-Vallée A. (2016). Social support and protection from depression: systematic review of current findings in Western countries. *Br. J. Psychiatry* 209 284–293. 10.1192/bjp.bp.115.169094 27445355

[B13] Gelaye B. Rondon M. B. Araya R. Williams M. A. (2016). Epidemiology of maternal depression, risk factors, and child outcomes in low-income and middle-income countries. *Lancet Psychiatry* 3 973–982. 10.1016/S2215-0366(16)30284-X 27650773 PMC5155709

[B14] Hwang I. C. Ahn H. Y. Choi S. J. (2021). Association between handgrip strength and mental health in Korean adolescents. *Fam. Pract.* 38 826–829. 10.1093/fampra/cmab041 34089052

[B15] Kavetsos G. (2011). “Chapter 11: Physical activity and subjective well-being: an empirical analysis,” in *The Economics of Sport, Health and Happiness*, eds Rodríguez P. Késenne S. Humphreys B. R. (Cheltenham: Edward Elgar Publishing).

[B16] Kjaer M. (1992). Regulation of hormonal and metabolic responses during exercise in humans. *Exerc. Sport Sci. Rev.* 20 161–184. 10.1249/00003677-199200200-00006 1623885

[B17] LaVigne T. Smith A. Shoulberg E. Bukowski W. (2016). Associations between physical fitness and children’s psychological well-being. *J. Clin. Sport Psychol.* 10 32–47. 10.1123/jcsp.2014-0053

[B18] Levis B. Benedetti A. Thombs B. D. (2019). Accuracy of patient health questionnaire-9 (PHQ-9) for screening to detect major depression: individual participant data meta-analysis. *BMJ* 365:l1476. 10.1136/bmj.l1476 30967483 PMC6454318

[B19] Liu C-j Shiroy D. M. Jones L. Y. Clark D. O. (2014). Systematic review of functional training on muscle strength, physical functioning, and activities of daily living in older adults. *Eur. Rev. Aging Phys. Act.* 11 95–106. 10.1007/s11556-014-0144-1

[B20] Lubans D. R. Cliff D. P. (2011). Muscular fitness, body composition and physical self-perception in adolescents. *J. Sci. Med. Sport* 14 216–221. 10.1016/j.jsams.2010.10.003 21111677

[B21] Moljord I. E. O. Moksnes U. K. Espnes G. A. Hjemdal O. Eriksen L. (2014). Physical activity, resilience, and depressive symptoms in adolescence. *Ment. Health Phys. Act.* 7 79–85. 10.1016/j.mhpa.2014.04.001

[B22] Mücke M. Ludyga S. (2018). Influence of regular physical activity and fitness on stress reactivity as measured with the trier social stress test protocol: a systematic review. *Sports Med.* 48 2607–2622. 10.1007/s40279-018-0979-0 30159718

[B23] Ortega F. B. Silventoinen K. Tynelius P. Rasmussen F. (2012). Muscular strength in male adolescents and premature death: cohort study of one million participants. *BMJ* 345:e7279. 10.1136/bmj.e7279 23169869 PMC3502746

[B24] Plummer F. Manea L. Trepel D. McMillan D. (2016). Screening for anxiety disorders with the GAD-7 and GAD-2: a systematic review and diagnostic metaanalysis. *Gen. Hosp. Psychiatry* 39 24–31. 10.1016/j.genhosppsych.2015.11.005 26719105

[B25] Rhodes R. E. Janssen I. Bredin S. S. D. Warburton D. E. R. Bauman A. (2017). Physical activity: health impact, prevalence, correlates and interventions. *Psychol. Health* 32 942–975. 10.1080/08870446.2017.1325486 28554222

[B26] Rodriguez-Ayllon M. Cadenas-Sanchez C. Esteban-Cornejo I. Migueles J. H. Mora-Gonzalez J. Henriksson P. (2018). Physical fitness and psychological health in overweight/obese children: a cross-sectional study from the ActiveBrains project. *J. Sci. Med. Sport* 21 179–184. 10.1016/j.jsams.2017.09.019 29031643

[B27] Saunders T. J. Gray C. E. Poitras V. J. Chaput J. P. Janssen I. Katzmarzyk P. T. (2016). Combinations of physical activity, sedentary behaviour and sleep: relationships with health indicators in school-aged children and youth. *Appl. Physiol. Nutr. Metab.* 41 (6 Suppl. 3) S283–S293. 10.1139/apnm-2015-0626 27306434

[B28] Skrove M. Romundstad P. Indredavik M. S. (2013). Resilience, lifestyle and symptoms of anxiety and depression in adolescence: the Young-HUNT study. *Soc. Psychiatry Psychiatr. Epidemiol.* 48 407–416. 10.1007/s00127-012-0561-2 22872359

[B29] Smith J. J. Eather N. Morgan P. J. Plotnikoff R. C. Faigenbaum A. D. Lubans D. R. (2014). The health benefits of muscular fitness for children and adolescents: a systematic review and meta-analysis. *Sports Med.* 44 1209–1223. 10.1007/s40279-014-0196-4 24788950

[B30] Sun J. Liang K. Chi X. Chen S. (2021). Psychometric Properties of the Generalized Anxiety Disorder Scale-7 Item (GAD-7) in a large sample of Chinese adolescents. *Healthcare* 9:1709. 10.3390/healthcare9121709 34946435 PMC8701121

[B31] Wang D. X. M. Yao J. Zirek Y. Reijnierse E. M. Maier A. B. (2020). Muscle mass, strength, and physical performance predicting activities of daily living: a meta-analysis. *J. Cachexia Sarcopenia Muscle* 11 3–25. 10.1002/jcsm.12502 31788969 PMC7015244

[B32] Warburton D. E. R. Bredin S. S. D. (2017). Health benefits of physical activity: a systematic review of current systematic reviews. *Curr. Opin. Cardiol.* 32 541–556. 10.1097/HCO.0000000000000437 28708630

[B33] Werner-Seidler A. Perry Y. Calear A. L. Newby J. M. Christensen H. (2017). School-based depression and anxiety prevention programs for young people: a systematic review and meta-analysis. *Clin. Psychol. Rev.* 51 30–47. 10.1016/j.cpr.2016.10.005 27821267

[B34] Wiese C. W. Kuykendall L. Tay L. (2018). Get active? A meta-analysis of leisure-time physical activity and subjective well-being. *J. Posit. Psychol.* 13 57–66. 10.1080/17439760.2017.1374436

[B35] Yalcinkaya H. Ucok K. Ulasli A. M. Coban N. F. Aydin S. Kaya I. (2017). Do male and female patients with chronic neck pain really have different health-related physical fitness, depression, anxiety and quality of life parameters? *Int. J Rheum. Dis.* 20 1079–1087. 10.1111/1756-185X.12389 24810182

[B36] Yeatts P. E. Martin S. B. Petrie T. A. (2017). Physical fitness as a moderator of neuroticism and depression in adolescent boys and girls. *Pers. Individ. Dif.* 114 30–35. 10.1016/j.paid.2017.03.040

[B37] Yu W. Sun J. Wu Y. Chen S.-T. (2021). Muscle-strengthening exercise links with lower odds for depression in adolescents. *Int. J. Ment. Health Promot.* 23 277–288. 10.32604/IJMHP.2021.016153

[B38] Zasadzka E. Pieczyńska A. Trzmiel T. (2021). Correlation between handgrip strength and depression in older adults-a systematic review and a meta-analysis. *Int. J. Environ. Res. Public Health* 18:4823. 10.3390/ijerph18094823 33946502 PMC8124581

